# Ratiometric Quantification of Dissolved Molecular Oxygen in Microplates for Biochemical Assays Using Palladium Porphyrin Photoluminescence

**DOI:** 10.1021/acsomega.6c04384

**Published:** 2026-09-10

**Authors:** Dina Podolskiy, Christoph Plieth

**Affiliations:** Zentrum für Biochemie und Molekularbiologie, Universität Kiel, Am Botanischen Garten 9, Kiel 24118, Germany

## Abstract

Many biochemical processes involve the consumption and/or release of molecular oxygen (O_2_). O_2_-induced photoluminescence quenching of palladium-tetrapyrrol derivatives can be used to continuously measure O_2_ concentrations in biochemical reactions and during microbial growth in standard microtiter plates (MTPs). Palladium­(II)-5,10,15,20-(tetrapentafluorophenyl)-porphyrin (1; CAS 72076-09-6) and Palladium­(II)-5,10,15,20-(tetraphenyl)­tetrabenzoporphyrin (2; CAS 119654-64-7) are introduced with this study. Spectral analyses of both compounds revealed that photoluminescence quenching by O_2_ is not evenly distributed throughout all wavelengths and can therefore be used ratiometrically. Experimentally determined photoluminescence lifetimes are around 500 and 300 μs for 1 and 2, respectively. A simple protocol is disclosed on how to immobilize the indicators on the bottom of MTP wells to give transparent, dye-doped polymer layers. We propose a straightforward procedure for how photoluminescence data can be processed and calibrated in terms of O_2_ concentrations. Diverse applications are demonstrated and discussed, which include oxygen consumption and production by microorganisms as well as by enzymatically catalyzed biochemical reactions. Various aspects are critically considered, such as the dependence of O_2_ solubility on temperature and salinity, the diffusion of O_2_ across diverse phase boundaries, the unwanted O_2_ ingress into the reaction mixture, the oxygen binding capacity of the MTP plastic material, and the pH dependence of the sensor layer. The findings and methods presented here open up a wide variety of high throughput assays involving changes of dissolved O_2_ as measurands for biochemical and biological activity.

## Graphical abstract

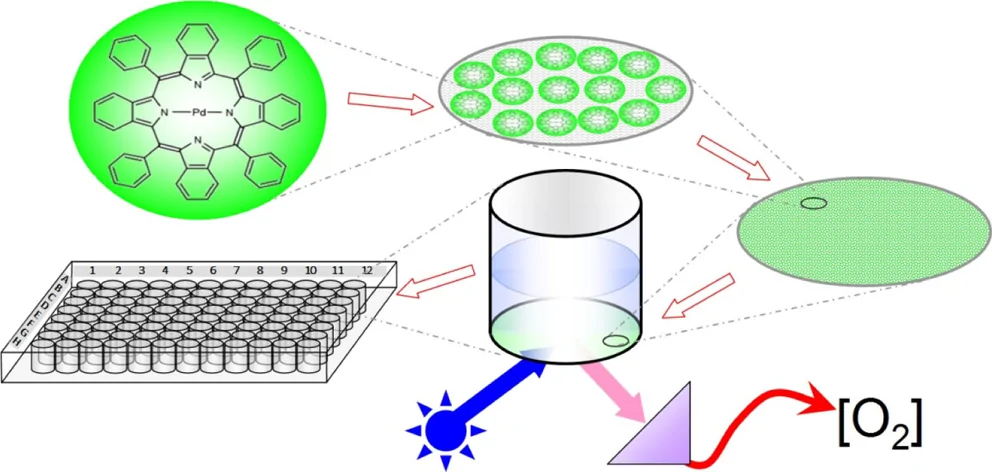

## Introduction

All life on earth is affected by O_2_, since abundant molecular oxygen (O_2_) influences almost all natural processes on our planet’s surface. Nearly 21% of our planet’s troposphere is O_2_, most of which has been generated by photosynthesis. It dissolves in water to a certain extent but also binds to and penetrates through diverse solid materials and surfaces.

In metabolism, O_2_ serves as an electron acceptor and is central for many biochemical redox processes. A prerequisite for this is that it dissolves in aqueous milieu. However, the solubility of oxygen in water is limited to concentrations of [O_2_] < 1 mM (<30 mg/L; for details, see DOTABLES^1–3^ ).

Oxygen consumption and production by living organisms are good indicators for metabolic processes like respiration^4^ and photosynthesis,^5–7^ respectively. Studying these processes requires precise methods to quantify [O_2_] and changes thereof in real time. In life science laboratories, two popular techniques are mainly in use. Both have advantages and limitations.^8,9^

First, amperometric electrodes exploit the effect that electrical currents, conducted by electrolyte solutions, are dependent on [O_2_].^6^ Clark-type oxygen electrodes are widely used in life science laboratories, as well as with water quality monitoring and field measurements.^10–12^ The output signal is linearly correlated with [O_2_].^13^ Hence, a simple two-point calibration can be used to convert the amperometric signal into an oxygen concentration.

Second, the photoluminescence (i.e., fluorescence and phosphorescence) of specific molecules can be quenched by O_2_.^14^ Devices exploiting this effect for [O_2_] quantification are called optodes or optrodes and are used with many biosensor applications.^9,15–17^ Noble metal porphyrins (NMPs) are widely used for these purposes.^18–22^ However, the development and production of specific biosensor opt­(r)­odes is not routine and requires some experience and engineering skills.^23–31^

Nowadays, microtiterplate (MTP) readers recording optical signals like absorbance, photoluminescence, or chemiluminescence belong to the standard equipment of many life science laboratories for high throughput (HTP) quantification of diverse parameters. Consequently, specific MTP assays have been developed, which allow the quantification of [O_2_] on the basis of photoluminescence quenching.^32,33^

In particular, phosphorescent NMPs are on the market for quantification of [O_2_] with fiber optics or in MTPs.^9,21,34^ However, because of some frame parameters, these optrode products are not widely established. They are costly, and some limitations such as undesirable oxygen ingress require attention and control experiments, which in turn require more of the material with increased expense.

The properties of any O_2_-optrode depend mainly on the quenching effect caused by O_2_, which depends on several factors such as phosphorescence lifetime, temperature, and diffusion rate of O_2_ within the sensor matrix and within the aqueous sample to be probed. In addition, the calibration, i.e., the conversion of phosphorescence signals from O_2_-optrodes into precise values of dissolved oxygen concentrations ([O_2_]), needs some consideration as O_2_ solubility depends on factors like temperature, salinity, and barometric pressure (Supporting Information. Temperature dependence of O_2_ solubility Figure SI 1, Salinity dependence of O_2_ solubility Figure SI 2).^1^

Photoluminescence emission from NMPs typically depends on both the concentration of the quencher molecule (here: O_2_) and the concentration of the NMP. Occasionally, the shape of the indicator’s spectrum changes with changing quencher concentration. This is equivalent to the fact that the indicator’s sensitivity to the quencher is wavelength-dependent. In the best-case scenario, a wavelength can be found in the spectrum where light emission is independent of the quencher concentration. This is called the iso-emission point, which is an intrinsic reference and only dependent on indicator concentration. Readings of emission intensities at wavelengths left and right of an iso-emission point can be used to calculate an emission ratio. The ratio then correlates with the quencher concentration, but is independent of indicator concentration. If this ratiometric approach is not feasible, a second quencher-independent dye can be added to have an extrinsic reference for ratioing. Typically, rhodamine dyes are used for this purpose.^21,38,39^ However, the mixture of both (indicator and extrinsic reference) may vary from batch to batch, with the result that data from different batches are less comparable. Therefore, intrinsically ratioable indicators are preferable.

The two indicators introduced here (Figure 1) are in the following named Pd-TFPP (1) and Pd-TPTBP (2) for short. We present the relevant spectral characteristics of 1 and 2 and provide a simple protocol on how to immobilize them in standard transparent polystyrene microtiter plates (PS-MTPs) for ratiometric quantification of [O_2_] in a plate reader using the fluorescence bottom-read option. We propose a straightforward calibration procedure to calculate [O_2_] from ratiometric phosphorescence data, thereby taking the dependence of the O_2_ solubility on temperature and salinity into account.

**Figure 1:**
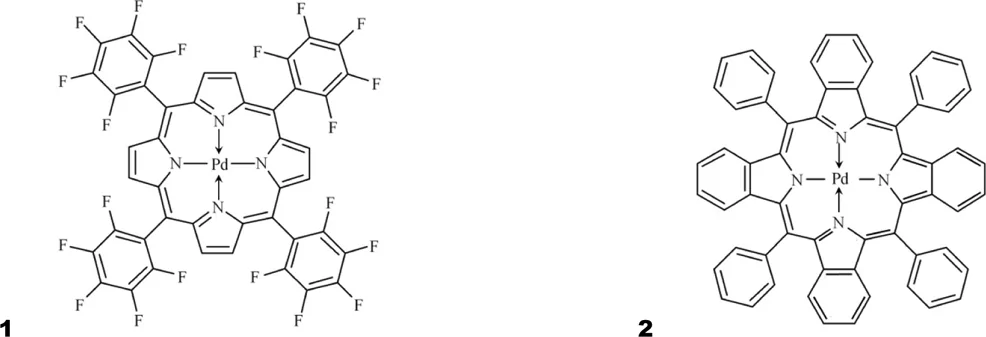
Structures of noble metal porphyrins (NMPs) used as O_2_ indicators for experiments presented below Palladium­(II)-5,10,15,20-(tetrapentafluorophenyl)­porphyrin (1 = Pd-TFPP; CAS 72076-09-6) M_W_ = 1079 g/mol and Palladium­(II)-5,10,15,20-(tetraphenyl)­tetrabenzoporphyrin (2 = TPTBP; CAS 119654-64-7) *M*_W_ = 919 g/mol.

As a proof of concept, we demonstrate how specific biochemical reactions involving consumption or production of O_2_ can be characterized. In addition, we employed bacteria and microalgae to show how their consumption and production of O_2_ could be quantified under diverse conditions. Finally, we provide detailed information on how to deal with interfering frame parameters, such as undesired O_2_ ingress.

## Results and Discussion

### Absorbance Spectra and Appearance of the O_2_ Indicators

Absorbance spectra (Figure 2) of both NMPs (1 and 2; Figure 1) appear as typical metalloporphyrin spectra with a Soret band in the blue region and two Q-Bands at longer wavelengths, namely, the vibronic (Q_1_) and the nonvibronic band (Q_0_).^35–37^ Key parameters of both spectra are summarized in Table 1.

**Figure 2:**
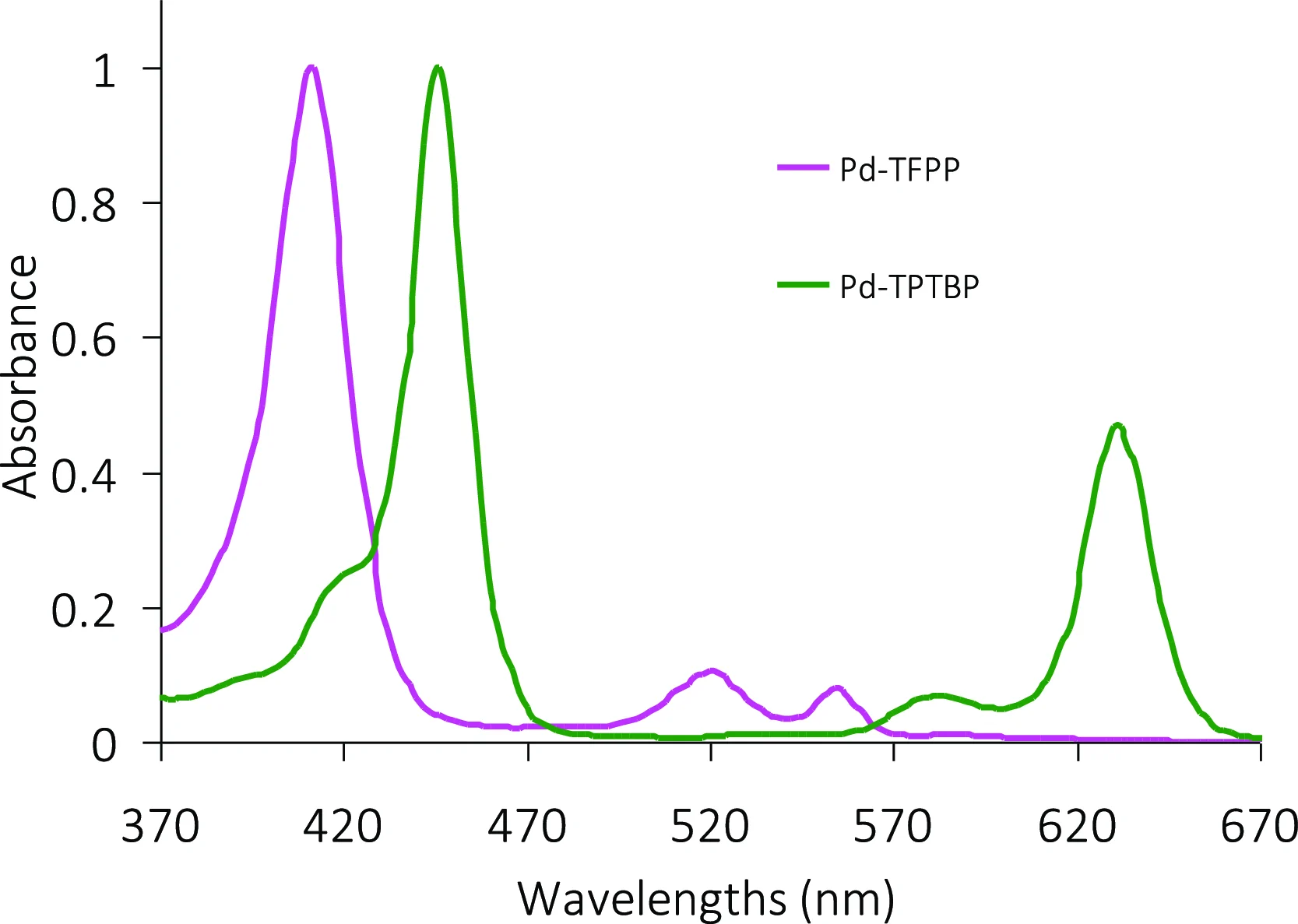
Absorbance spectra of 1 and 2 MTP wells doped with Pd-NMPs, as described in the experimental section. Absorbance of the indicator layer was scanned by a plate reader, and spectra were normalized by the peak of their Soret band. Pd-TFPP (1) absorbs mainly in the blue and green regions and thus appears red, while Pd-TPTBP (2) absorbs in the blue and the red region, having a distinct green absorption gap and thus appears, like chlorophyll, green. Spectral characteristics of the peaks are summarized in Table 1.

**Table 1:** Absorbance Characteristics

	Pd-TFPP (1)	Pd-TPTBP (2)
Soret band peak (fwhm)	412 nm (26 nm)	446 nm (20 nm)
Vibronic Q1-band peak (fwhm)	520 nm (27 nm)	582 nm (30 nm)
Peak height relative to Soret	0.10	0.07
Nonvibronic Q0-band peak (fwhm)	554 nm (16 nm)	632 nm (22 nm)
Peak height relative to Soret	0.08	0.47

Both, 1 and 2, absorb visible light. 1 appears red-pink (Supporting Information Figure SI 1: Flat-bottom MTPs for [O_2_] quantification) because it absorbs mainly in the blue region around 412 nm (Figure 2), whereas 2 has two absorbance peaks in the visible range at 446 and 632 nm and thus, like chlorophyll, appears green (Figure SI 1). The main difference in color is caused by a lack of absorbance in the 600 nm range, as seen in the spectrum of 1 (Figure 2).

### Photoluminescence Spectra

The excitation spectra of 1 and 2 (Figures 3A and 4A) are similar in shape when compared with their absorption spectra (Figure 2), indicating that absorbed light is converted to phosphorescence at all wavelengths and heat dissipation (i.e., vibronic excitation) is negligible.

**Figure 3:**
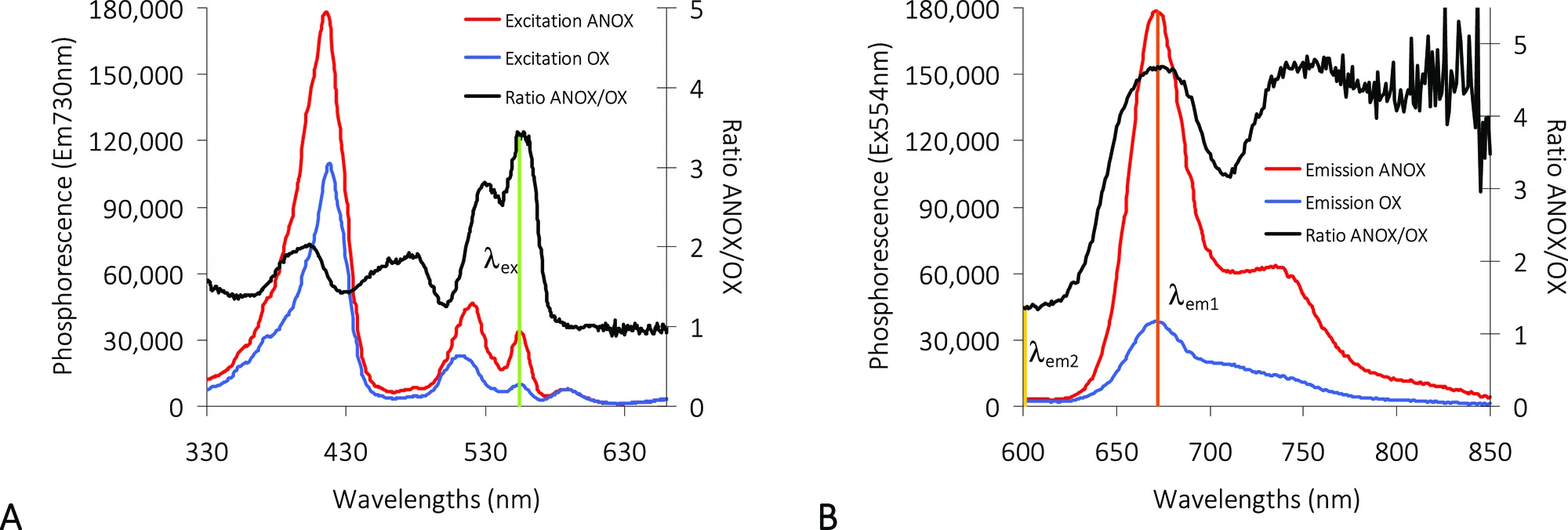
Phosphorescence spectra of Pd-TFPP (1) under oxygenated conditions (blue lines; wells filled with H_2_O) and anoxic conditions (red lines; wells filled with 2 M Na_2_SO_3_ solution). Black lines indicate the ratio of the ANOX data divided by the OX data calculated for each wavelength. Phosphorescence is given in arbitrary units. Spectra are averages of *n* = 12 replicates. (A) Excitation spectra taken with emission at λ_em_ = 730 nm; (B) emission spectra taken with excitation at λ_ex_ = 554 nm.

**Figure 4:**
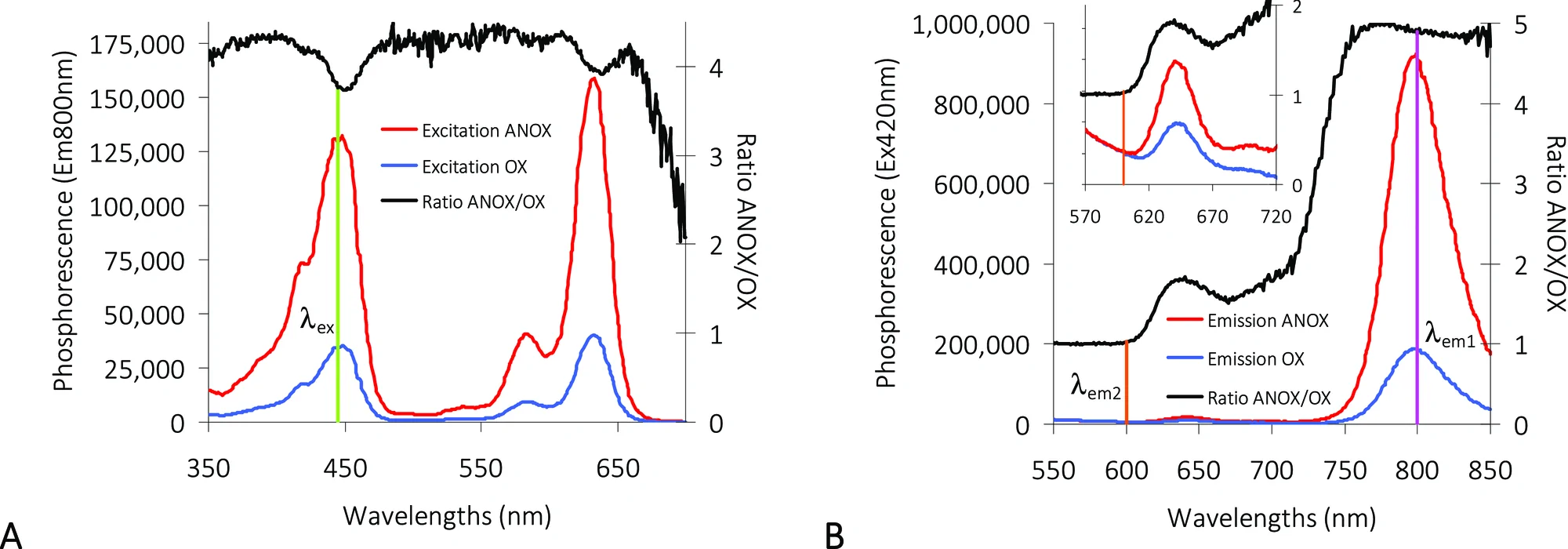
Phosphorescence spectra of Pd-TPTBP (2) under oxygenated conditions (blue lines; wells filled with H_2_O) and anoxic conditions (red lines; wells filled with 2 M Na_2_SO_3_ solution). Black lines indicate the ratio of the ANOX data divided by the OX data calculated for each wavelength. Phosphorescence is given in arbitrary units. Spectra are averages of *n* = 12 replicates. (A) Excitation spectra taken with emission at λ_em_ = 800 nm; (B) emission spectra taken with excitation at λ_ex_ = 420 nm.

However, the indicators’ O_2_ sensitivities are not evenly distributed throughout all wavelengths. This is demonstrated by the ratio spectra (black lines in Figures 3 and 4). The ratio of phosphorescence emission intensities from O_2_-low and O_2_-rich wells varies and reaches values of >4. Higher ratios are not reached here, due to the incomplete deprivation of [O_2_] by Na_2_SO_3_ and the temperature-dependent limited O_2_ solubility in aqueous solutions (Supporting Information Figure SI 2: Temperature dependence of O_2_ solubility).

### Characteristics of the Phosphorescence Spectra

The recognition of wavelength-dependent quenching and of spectral shifts upon quencher collision or ligand binding is possible when spectra are normalized by their area. If the quenching effect was evenly distributed, then normalized spectra taken under O_2_-saturated conditions (OX) would be identical with normalized spectra taken under low [O_2_] (ANOX). Hence, the difference between both normalized spectra (designated “MiniMax spectrum”; Figure 5) would be constant null. However, as shown in Figure 5, the normalized emission spectra reveal wavelength-dependent quenching.

**Figure 5:**
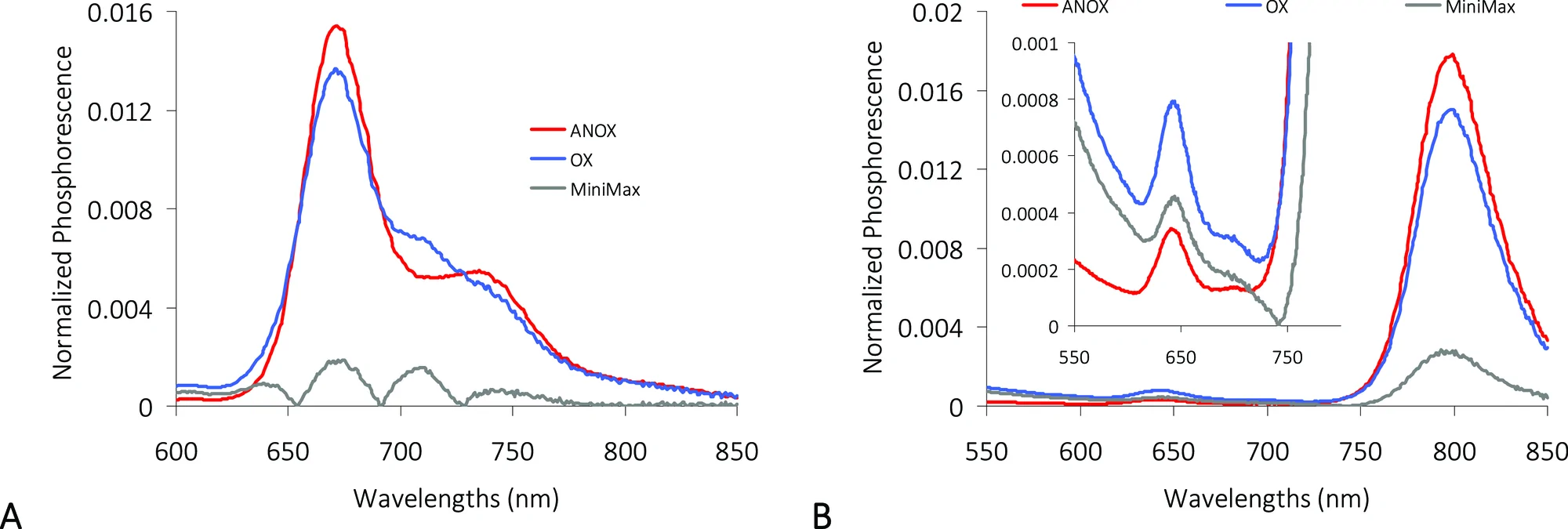
Phosphorescence emission spectra normalized by the area under the curve (*A* = 1). MiniMax spectra (gray lines) are the absolute difference between ANOX and OX. (A) Normalized spectra of 1 calculated from spectra shown in Figure 3B. The nulls of the MiniMax spectrum reveal isoemission (“isosbestic”) points at 655, 692, and 729 nm. (B) Normalized spectra of 2 calculated from spectra in Figure 4B. An isoemission point obtained from the null of MiniMax is at 744 nm. All spectra are averages of *n* = 12 technical replicates.

When photoluminescent indicators are quenched equally at all wavelengths, then a quencher-independent fluorophore is required as an internal standard for (pseudo)­ratiometric measurements. For Pt-NMPs as oxygen indicators, Rhodamine was used as a reference standard to enable ratiometry.^21,38,39^ However, when phosphorescence quenching is not equally distributed over all wavelengths, as is the case here (Figure 5A,B), then two different phosphorescent species can be assumed in the sample. The spectrum obtained is then an overlay of their distinct spectra. This effect becomes evident with the data set shown in Figure 5. The discontinuous minima (null points) of the difference spectrum (“MiniMax”; gray lines in Figure 5) reveal the isoemission points. These iso-points separate the wavelength range for measuring the [O_2_], from wavelengths that can serve as an internal reference. They make it evident that emission intensities at different wavelengths can be used for ratiometric measurements.

### Selecting Appropriate Wavelengths for [O_2_] Measurements

Finding the optimal wavelengths for [O_2_] measurements requires a compromise between maximum phosphorescence intensity and O_2_ sensitivity.

With Pd-TFPP (1), the maximum O_2_ sensitivity in excitation is given with the Q_0_-band at 554 nm (Figure 3A), although maximum phosphorescence can be achieved with excitation in the Soret band around λ_ex_ = 412 nm. Considering phosphorescence emission, peak phosphorescence and maximum O_2_ sensitivity are at λ_em1_ = 672 nm (Figure 3B). At λ_em2_ = 600 nm, emission phosphorescence is almost O_2_-insensitive (ratio ANOX/OX ≈1). This suggests that this emission wavelength would be suitable as a reference for ratioing (Table 2).

**Table 2:** Excitation and Emission Wavelength**s**

The phosphorescence intensity ratios R = F_λem1_/F_λem2_ were used as indicator variables for dissolved O_2_. Wavelengths have been chosen according to phosphorescence yields and O_2_ responsiveness and are used for all experiments described in this study. The numerator wavelength λ_em1_ for ratioing is at maximum emission. The denominator wavelength, λ_em2_, is where low or no phosphorescence quenching is seen (i.e., ratio ANOX/OX ≈ 1; Figures 3 B, 4 B)					
Pd-TFPP (1)			Pd-TPTBP (2)		
λ_ex_	λ_em1_	λ_em2_	λ_ex_	λ_em1_	λ_em2_
554 nm	672 nm	600 nm	445 nm	800 nm	600 nm

The situation is different with Pd-TPTBP (2). Maximum phosphorescence emission and maximum O_2_ sensitivity are around λ_em1_ = 800 nm (Figure 4B). O_2_ insensitivity with still reasonable phosphorescence intensity is seen with λ_em2_ = 600. When choosing these two wavelengths for ratioing, neither the Q_0_ nor Q_1_ band can be used for excitation. Hence, excitation in the Soret band at λ_ex_ = 445 nm is required to achieve acceptable phosphorescence intensities (Figure 4A). The wavelengths for phosphorescence emission ratioing (R = F_λem1_/F_λem2_) and [O_2_] calculation chosen for all further experiments described below are summarized in Table 2.

### Phosphorescence Lifetimes and Adjustment of Integration times

The choice of an appropriate integration time *T* for photoluminescence measurements is important, because it affects both the maximum possible sampling rate and the signal-to-noise ratio. After excitation, the population of excited states decays exponentially and gives off light with a lifetime (i.e., time constant) of τ according to eq 1.

1 $$F\left(t\right)=F_{0}\cdot e^{-t/\tau }$$

Phosphorescence lifetimes τ of NMPs are typically in the range between 20 and 2000 μs.^18,40–43^ Direct lifetime measurements are not possible with a plate reader. However, the instrument allows a precise setting of the duration of photon counting T (i.e., integration time) and thereby performs a multiwindow method.^43^ Thus, NMP phosphorescence photons collected with various integration times *T* at fixed gain settings provide a means of measuring the lifetime τ. The amount of light emitted during the decay process (eq 1) is determined by *T* (eqs 2).

2a $$\int_{0}^{T}F\left(t\right)dt={\left[-\tau \cdot F_{0}\cdot e^{-t/\tau }\right]}_{0}^{T}$$

2b $$\Rightarrow S\left(T\right)=F_{0}\cdot \tau \cdot \left(1-e^{-T/\tau }\right)$$

This integral *S*(*T*) is a function of *T* with lifetime τ as a parameter (eq 2b).

A series of integration times *T* from 20 to 2000 μs gave data sets *S*(*T*), as shown in Figure 6. Fitting Chapman model functions (eq 3) to such data with the exponent c fixed to *c* = 1 allowed parameters *a* and *b* to be determined.

3 $$chapman\;model\;function\;y\left(x\right)=a\cdot {\left(1-e^{-b\cdot x}\right)}^{c}$$

**Figure 6:**
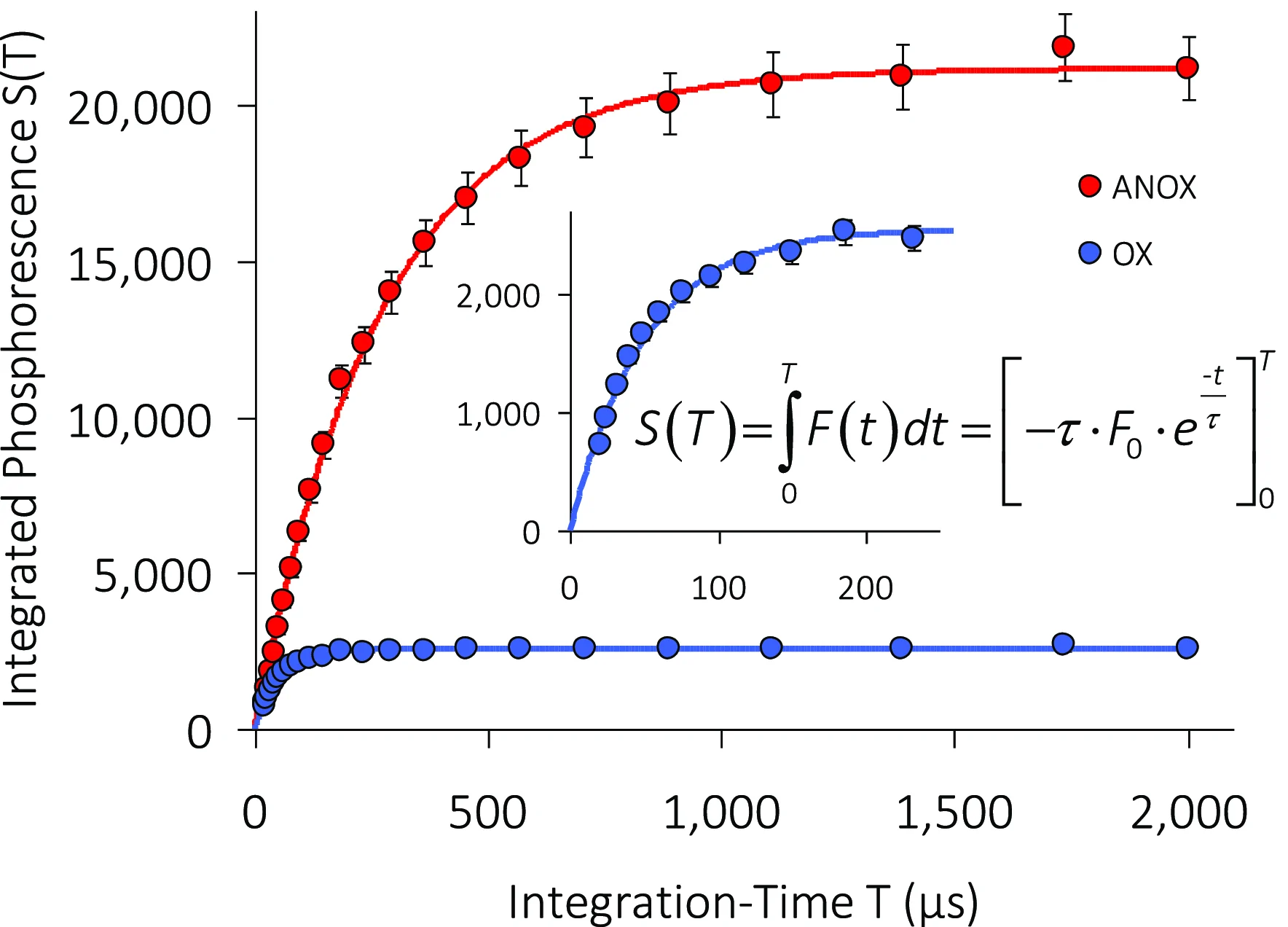
Integrated phosphorescence S from Pd-TPTBP (2) plotted over integration time *T* demonstrates how phosphorescence lifetimes τ under anoxia versus oxygenated conditions were obtained. A series of phosphorescence S (λ_ex_ = 445 nm; λ_em_ = 800 nm) were collected with increasing integration times *T* (20 to 2000 μs). MTP wells were filled either with an O_2_ absorber and sealed (red line; oxygen-free samples) or with H_2_O and left open (blue line; oxygenated samples). A Chapman model function was fitted to the data to obtain the parameters giving S­(T). Lifetimes τ were calculated, as described in the main text (eqs 2–4). Data are averages of n = 12. Error bars represent SD. SD is below symbol size where error bars cannot be seen.

From a and b, the parameters τ and *F*_0_ of *S*(*T*) could be calculated (eqs 4a,b)

4a $$\tau =\frac{1}{b}$$

4b $$F_{0}=a\cdot b$$

Using this method, lifetimes τ of 1 and 2 were determined, as listed in Table 3.

**Table 3:** Phosphorescence Lifetimes τ

lifetimes τ are calculated from integrated phosphorescence measurements *S*(*T*) (eq 2b)^a^						
Pd-TFPP (1)				Pd-TPTBP (2)		
Temp. (degC)	τ ANOX (O_2‑_free)	τ OX (O_2_-saturated)	Stern–Volmer quotient τ_0_/τ	τ ANOX (O_2_-free)	τ OX (O_2_-saturated)	Stern–Volmer quotient τ_0_/τ
25	503 μs (±28)	54 μs (±2.3)	9.35 (±0.5)	312 μs (±13)	48 μs (±1.4)	6.55 (±0.15)
30	510 μs (±39)	52 μs (±3)	9.88 (±0.78)	294 μs (±16)	47 μs (±1)	6.31 (±0.24)
37	471 μs (±66)	48 μs (±3)	9.89 (±1.01)	295 μs (±4)	45 μs (±2)	6.55 (±0.2)

a Values given are averages of *n* ≥ 5 experiments. Numbers (±) in parentheses denote SD.

The data (Table 3) show that lifetimes are shorter in the presence of oxygen, proving dynamic quenching. Values are in the same range as reported elsewhere.^18,40–43^ Thus, for NMPs, integration times in the four-digit range of μs should be chosen for phosphorescence recordings. For all further experiments presented here, the plate reader was set to an integration time *T* of 1000 μs.

The rate of collisional quenching typically increases with increasing temperatures. This effect should become apparent by a decrease in τ. Here, experiments were performed at different temperatures (25 °C; 30 and 37 °C) but gave no significant changes in τ (Table 3). This is understandable, because the phosphorescent indicators are immobilized in a PS layer and the diffusion of oxygen into and within the plastic material is limited when compared with its mobility in aqueous solutions or in air.^44–46^

### Quantification of Oxygen Ingress

Under ambient conditions, aqueous solutions in contact with the atmosphere contain an O_2_ concentration of a few hundred micromolar (100 μM < [O_2_] < 500 μM) when in equilibrium (Supporting Information Figure SI 2; DOTABLES).^3^ O_2_ can diffuse from the gas phase into the solution (ingress) and vice versa (egress). In the presence of sulfite (Na_2_SO_3_), dissolved oxygen is consumed, and sulfite is converted to sulfate (Na_2_SO_4_) (Reaction 1). Cobalt, as a catalyst, accelerates this process.^47^ Thus, a solution with a sufficiently high Na_2_SO_3_ concentration and a few ppm of CoCl_2_ is low in [O_2_].
R1

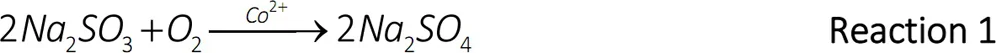




Wells with immobilized Pd-NMPs were filled with solutions containing various concentrations of sulfite ([Na_2_SO_3_]). Pd-porphyrin phosphorescence was recorded over several hours during which the wells remained unsealed, allowing O_2_ ingress from the atmosphere.

Phosphorescence remains high as long as all incoming O_2_ is instantly compensated by sulfite-mediated O_2_ consumption (Reaction 1). However, as soon as all sulfite is converted to sulfate, quenching of phosphorescence starts rapidly (Figure 7A). The time points at which the Na_2_SO_3_ in the wells has been consumed by incoming O_2_ are marked by the minimum values of the derivatives (d*P*/d*t*) of the data (Figure 7B).

**Figure 7:**
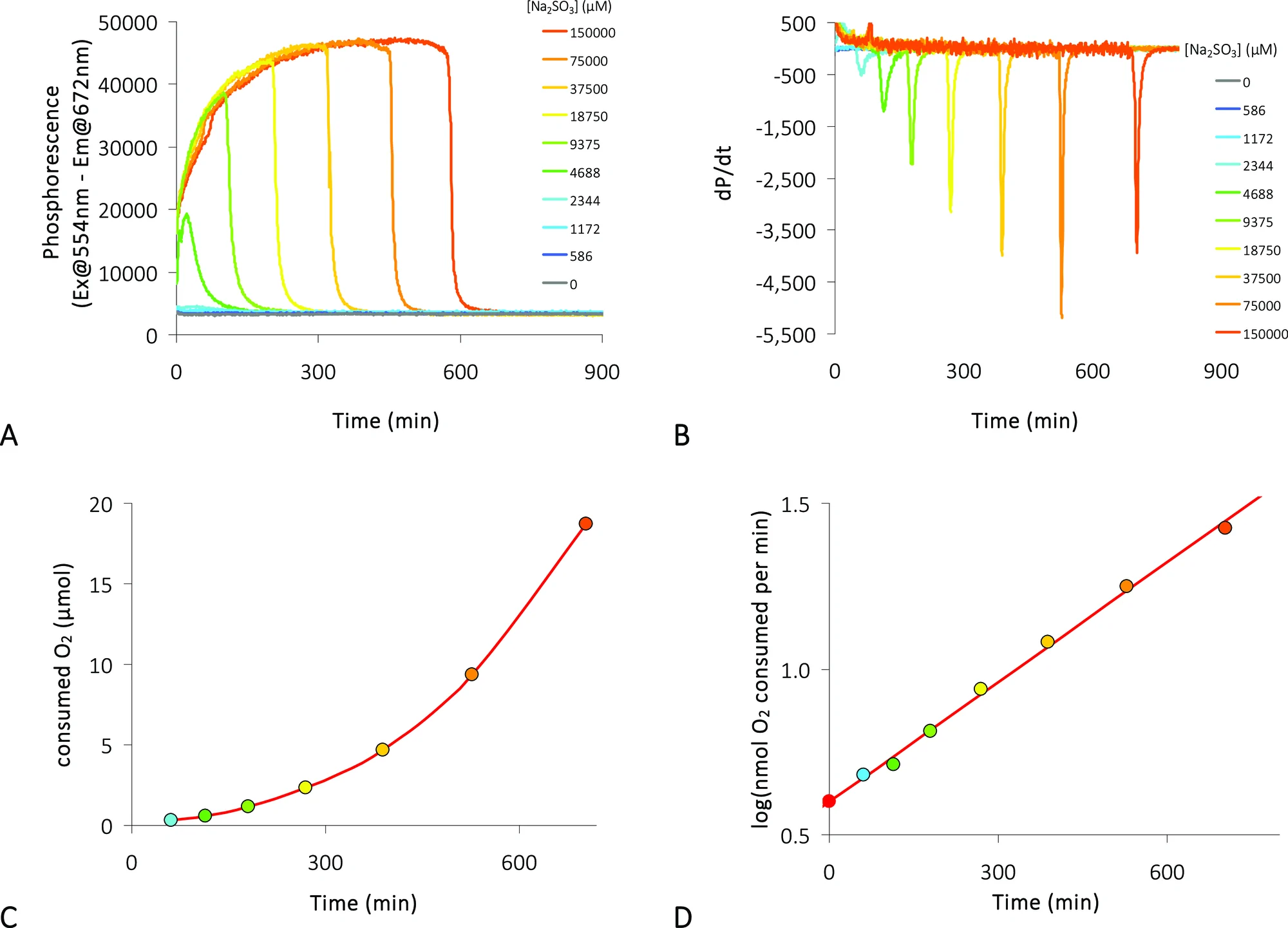
Oxygen ingress quantified using dilution series of Na_2_SO_3_. (A) Pd-TFPP-coated MTP loaded with 200 μL of diverse concentrations of Na_2_SO_3_ was exposed to ambient air, and phosphorescence (λ_ex_ = 554; λ_em_ = 672 nm) was recorded. Sample rate = 1·min^–1^. (B) The time derivatives calculated from data presented in A reveal, with their downward peaks, the time points (i.e., consumption times) where all Na_2_SO_3_ (sulfite) is converted to Na_2_SO_4_ (sulfate). (C) The consumed amount of O_2_ (in μmol) as calculated from the converted amount of Na_2_SO_3_ (Reaction 1) plotted over time reveals an exponential dependence. (D) The log of oxygen consumption [in nanomol O_2_ per time (min)] calculated from data shown in (C) plotted over time gives a straight line. Extrapolation of the line intersects with the ordinate (red dot at *y* = 0.6). This intersection marks the oxygen ingress/egress equilibrium (here 4 nmol/min) if no Na_2_SO_4_ was in the solution.

The amount of O_2_ needed to convert the sulfite can be calculated from the amount of sulfite the well was loaded with (stoichiometry: 1 O_2_ per 2 Na_2_SO_3_; Reaction 1). The amount of consumed O_2_ plotted against time reveals an exponential increase (Figure 7C), showing that in this process, the consumed O_2_ and time are not linearly correlated but rather follow an exponential dependence. Thus, in a semilogarithmic plot of log­(consumed O_2_/time) against time, a straight line is obtained (Figure 7D). The extrapolation of the trend to the *y*-axis reveals the oxygen ingress if there was no Na_2_SO_3_ in solution. This gives the O_2_ ingress rate, which at equilibrium is equal to O_2_ egress.

Intuitively, a doubling of [Na_2_SO_3_] should lead to a doubling of the time needed to convert all sulfite into sulfate. However, the whole process is driven by diffusion, which, in turn, is dependent on concentration gradients (Fick’s laws). The high diffusion rate of O_2_ in the gas phase leads to a quick depletion of sulfite at the solution surface. With increasing [Na_2_SO_3_], steeper vertical concentration gradients of Na_2_SO_3_ are established in the solution. This causes accelerated diffusion in the liquid phase, disproportionately faster turnover (Reaction 1), and shorter consumption times *t*_c_.

### Proof of the Calibration Linearity

Since dynamic phosphorescence quenching is assumed as described by the Stern–Volmer equation,^48^ a uniform quenching would be expected at all phosphorescence wavelengths. However, the NMPs (1, 2) used here show a wavelength-dependent quenching by O_2_ (Figures 3 and 4). This raises the possibility that there is no pure dynamic quenching process at work, which may possibly result in a nonlinear (e.g., logistic or sigmoidal) relationship between phosphorescence ratios R and [O_2_].

We addressed this concern and recorded [O_2_] amperometrically in parallel to the ratiometric method (Supporting Information Figure SI 4: Correlating NMP phosphorescence ratios with amperometric oxygen recording). An amperometric signal is always linearly correlated with [O_2_], provided the O_2_ electrode is properly prepared.^10,49^ If a nonlinear relationship exists between NMP phosphorescence ratio R and [O_2_], this would become apparent when comparing the optical signal R with the simultaneously recorded amperometric signal. Since the data confirm linearity (Supporting Information Figures SI 4B,C—insets), it is evident that the linear calibration used (eqs 8–11 in the Methods section) is appropriate.

### Adjustments for Temperature

The temperature dependence of O_2_ solubility needs to be considered when [O_2_] is recorded with varying temperature. During phosphorescence measurements, the plate reader records the temperature (in °C) inside the instrument. These temperature data can be used for calibration correction according to eq 7 (Methods section below).

When calibration is performed without temperature correction (eq 5), then a constant [O_2_] at all temperatures probed is the result (Figure 8 light symbols). However, this does not correspond to the actual [O_2_], which decreases with increasing temperatures due to the decreased O_2_ solubility^3^ (expressed as [O_2_]_max_) and the resulting O_2_ egress. Thus, with variations in temperature during the experiment, a temperature-dependent correction, as given by eq 7, is necessary and leads to more reliable results (Figure 8, dark symbols).

**Figure 8:**
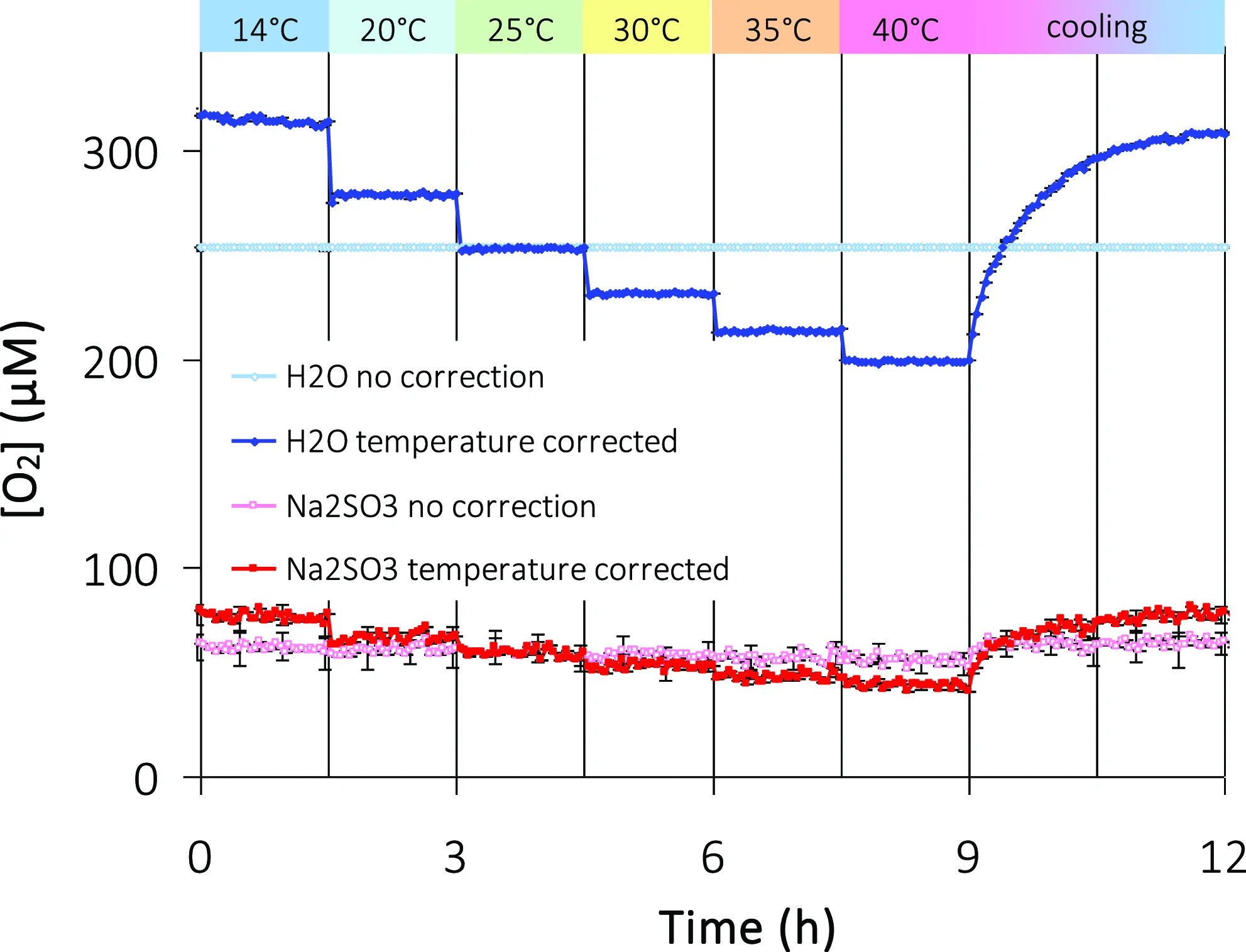
Necessity of temperature correction with ratiometric [O_2_] recording. Wells were filled either with plain water (blue symbols) to achieve [O_2_]_max_ or with a sulfite solution (red symbols) to adjust to a relatively low [O_2_]. [O_2_] appears to be constant at all temperatures (light symbols) when phosphorescence ratios were converted to [O_2_] using eq 5, with [O_2_]_max_ = 254 μM (at 25 °C), thereby disregarding the temperature dependence of O_2_ solubility. Implementing the temperature correction of [O_2_]_max_ (eq 7) leads to reliable results (dark symbols) showing that [O_2_] decreases with increasing temperature, because reduced solubility causes O_2_ egress. Experimental conditions: 200 μL per well; sample rate = 1/3·min^–1^. [Na_2_SO_3_] = 2M; *R*_max_ was taken from wells filled with O_2_-absorber powder. Average of *n* = 12; error bars represent SD.

### Quantification of O_2_ Consumption and Production

Some enzymes, such as oxygenases or laccases, consume O_2_ when catalyzing a reaction.^50–53^ Their enzymatic activities can be quantified by monitoring the [O_2_] in the reaction mixture.^15,54^ Consequently, NMP phosphorescence in MTPs should be useful to characterize such enzymes, to detect, and to quantify their substrates.

### O_2_ Consumption by the Laccase Reaction

The laccase reaction presented here is an example of enzymatically catalyzed O_2_ consumption.

The enzyme converts pyrogallol to pupurogallin (Reaction 2) with an optimum at pH = 5.5.^55^

R2

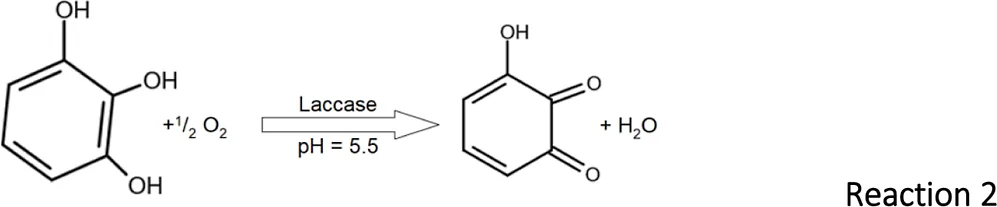




The data presented in Figure 9 reveal that a characterization of enzyme turnover is possible. However, interfering processes, such as O_2_ ingress, must be kept in check (details below).

**Figure 9:**
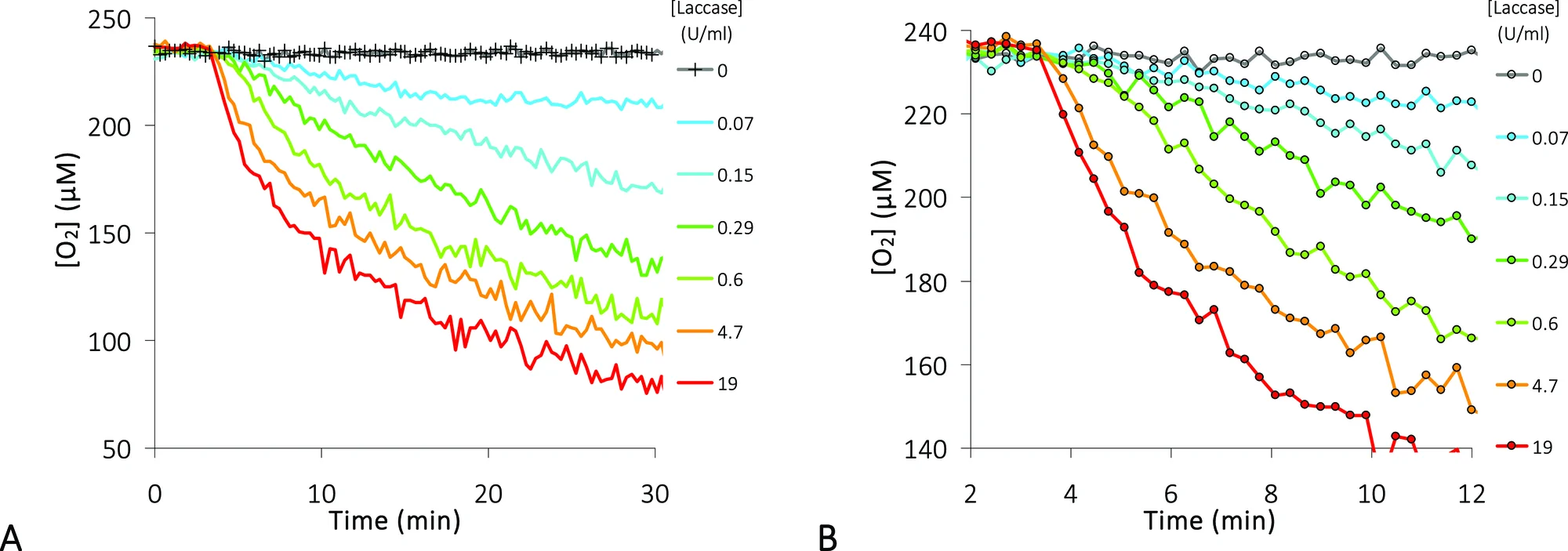
Consumption of O_2_ by different laccase activities recorded ratiometrically using Pd-TPTBP (2) phosphorescence. (A) The reaction was started at *t* = 3.3 min by injection of pyrogallol into a reaction milieu, and wells were sealed immediately. (B) Enlargement of a data section from (A). Final assay conditions: CPB pH = 5.5, [Pyrogallol] = 6 mM; 28.2 °C; Salinity 5.1 mS/cm; F_Sal_ = 0.984; [Laccase] as indicated in the inset legends; Sample rate = 1/18·s^–1^.

### O_2_ Consumption by Respiration during Bacterial Growth

Growth of and fermentation by aerobic microorganisms in batch cultures are restricted by nutrients and O_2_ supply. The effects of oxygen deficiency on bacterial growth can be monitored with optical oxygen sensors during the whole culture period when the optical density (OD_600_) of the culture is recorded in parallel. Pd-TPTBP significantly absorbs light in the range of 600 nm (Figure 2), whereas the absorption by Pd-TFPP in this range is negligible. This makes Pd-TFPP the better choice when monitoring cell density at 600 nm in parallel with [O_2_].

*Vibrio natriegens* is one of the fastest growing bacterial species. It can reach doubling times as low as 10 min under aerobic conditions.^56^ Here, *V. natriegens* was grown in clear MTPs coated with NMP in order to monitor cell density (OD_600_) and [O_2_] simultaneously (Figure 10). With foil-sealed wells, exponential growth started after a lag phase of ca. 2.6 h and O_2_ consumption started in parallel. Growth leveled off when [O_2_] dropped below 20 μM. Removal of the sealing foil, to allow for O_2_ ingress, caused further growth by *a* factor of 1.6 (OD_600_ increase from 0.8 to 1.3), indicating that growth retardation within the first 20 h was mainly restricted by the lack of gas exchange. The second growth period stopped at OD_600_ = 1.3 (ln­(OD_600_) = 0.26), suggesting that nutrients were exhausted. The O_2_ ingress seen during the last 12 h of the experiment (Figure 10, blue line) reflects the fact that respiration of the cells had ceased.

**Figure 10:**
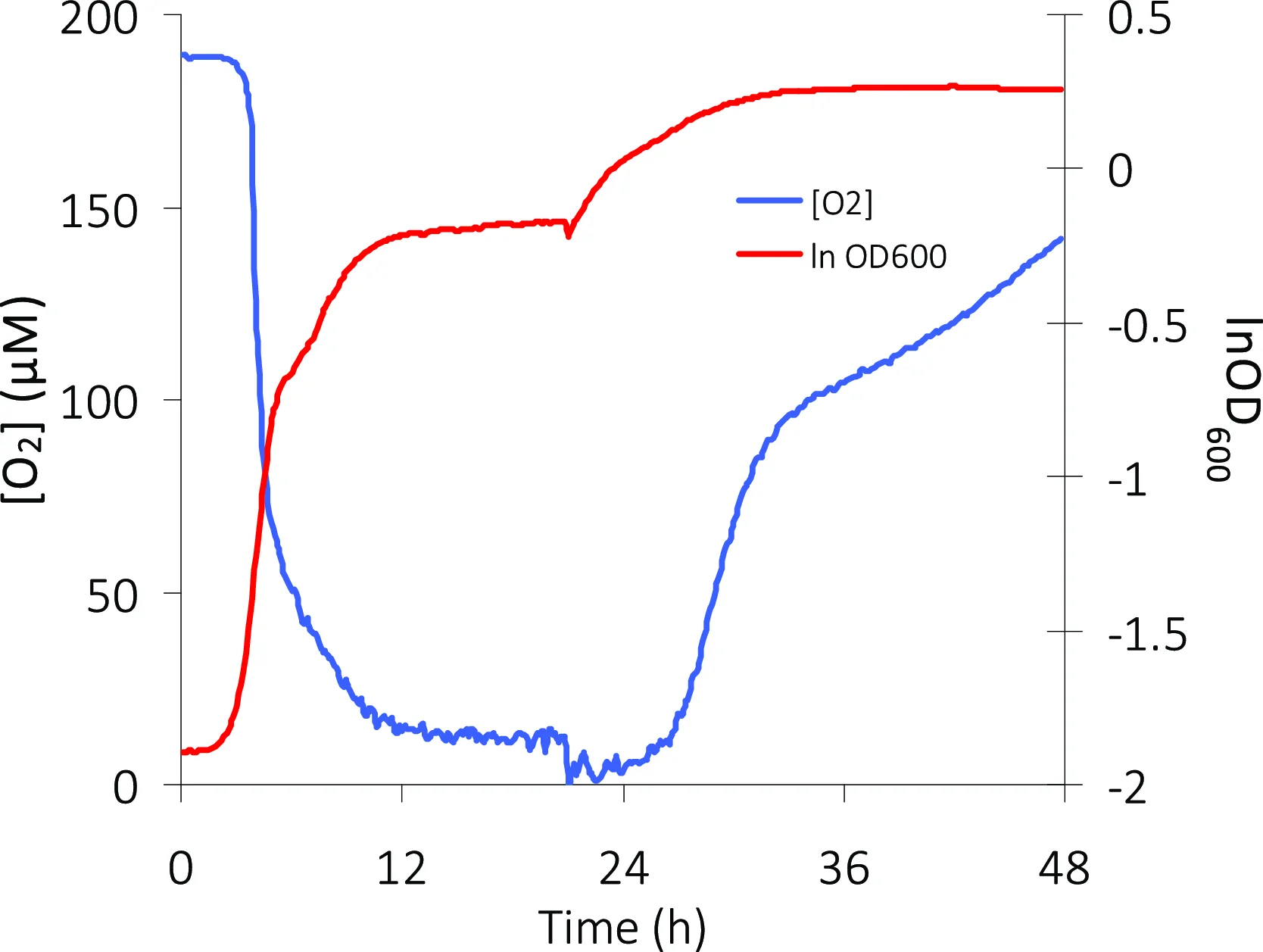
Growth and respiration of *Vibrio natriegens* monitored in MTP wells. The [O_2_] measured by Pd-TFPP phosphorescence ratiometry (left ordinate, blue line) was recorded in parallel to OD_600_ (right ordinate, logarithmic scale, red line). The maximum growth rate coincides with maximum respiration at *t* = 4.3 h. The sealing foil was removed at *t* = 21 h, allowing O_2_ ingress. This resulted in further growth, whereas growth retardation (from *t* = 27 h onward) leads to less O_2_ consumption and consequently increased O_2_ ingress. Culture conditions: LB + v2-medium; 28 °C; salinity 44 mS/cm; F_Sal_ = 0.85; linear shaking 1.5 mm; AVR of *n* = 33; SD < 12%; sample rate = 1/5 min^–1^.

It appears reasonable to assume that the O_2_ shortage is the cause of growth retardation when MTP wells are sealed and that growth resumes as soon as gas exchange is restored, provided nutritional resources are still available. However, there may be other decisive factors, such as pH and bicarbonate, that are affected by bacterial growth when gas exchange is blocked. If needed, pH could also be monitored by fluorescence ratiometry in MTPs using specific immobilized pH-sensitive fluorescein derivatives.^21^

### O_2_ Production by the Catalase Reaction

Catalase catalyzes the disproportionation of hydrogen peroxide (H_2_O_2_) (Reaction 3). The resulting [O_2_] increase is efficient and quick when H_2_O_2_ and catalase are combined (Figure 11). This allows us to estimate the response time of the embedded O_2_-indicator relative to the used sample rate.
R3

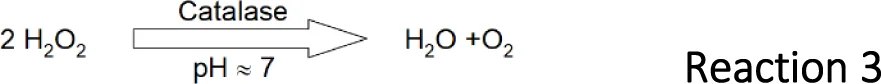




**Figure 11:**
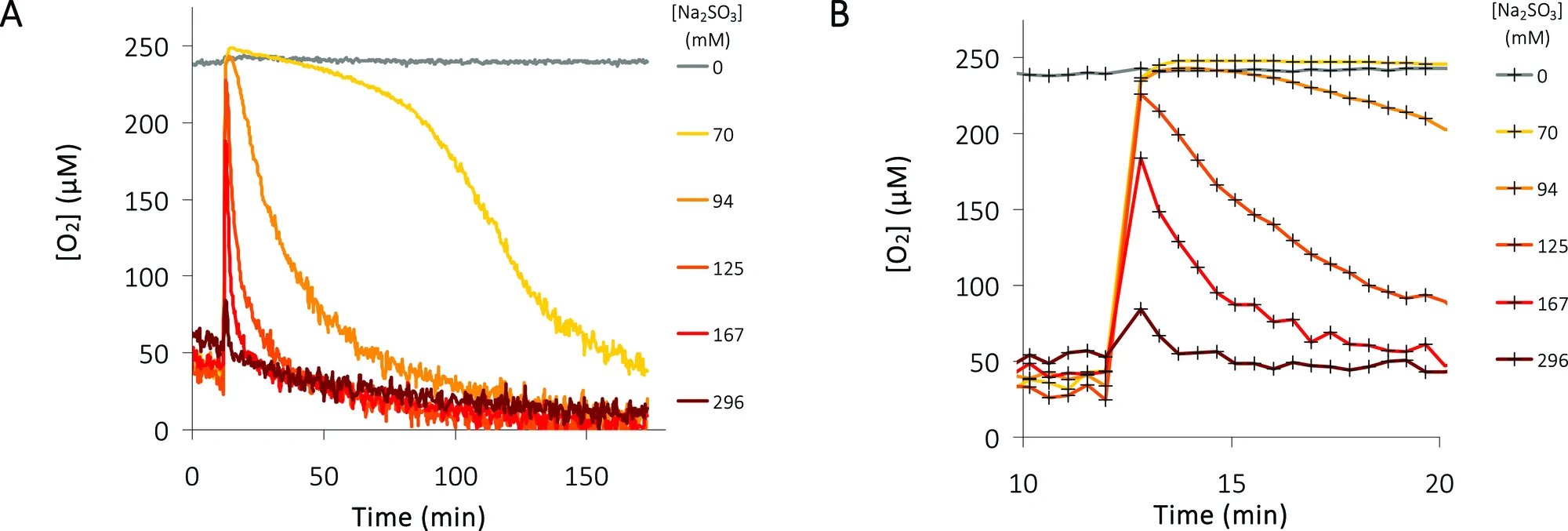
O_2_ bursts produced by catalase monitored in the presence of different sulfite concentrations. (A) The lower the [Na_2_SO_3_], the more pronounced is the catalase-induced O_2_ burst. The reactions were started at *t* = 12 min by injection of H_2_O_2_ into a sulfite solution containing catalase. [O_2_] was recorded ratiometrically with Pd-TPTBP (2). (B) The close-up indicates an instantaneous response of the indicator. Final assay composition: KPB 50 mM pH = 7; 28 °C; [H_2_O_2_] = 21 mM; [Na_2_SO_3_] indicated by inset legends; salinity (F_Sal_; eq 8) is taken into account. Sample rate ca. 2·min^–1^.

The experiment (Figure 11) demonstrates that the O_2_ bursts released by the catalase reaction (Reaction 3) are compensated (“buffered”) by Na_2_SO_3_ (Reaction 1) as long as [Na_2_SO_3_] is high enough. With high [Na_2_SO_3_], the free O_2_ in the medium remains largely unaffected by the catalase reaction (dark red line). However, with lower [Na_2_SO_3_], the released O_2_ causes noticeable perturbations of [O_2_] in the medium because the O_2_ consumption by Na_2_SO_3_ is slower than O_2_ production by catalase. The indicator shows a rapid response relative to the data sampling frequency and immediately displays the change in [O_2_] without delay. From this, it can be concluded that the coating is thin enough (thickness of indicator film ≈1.5 μm) to ensure quick response, although the diffusion of O_2_ is more than 100 times slower in the polystyrene film (*D* = 1 · 10^–7^ cm^2^·s^–1^)^57^ when compared with water (*D* ≈2 · 10^–4^ cm^2^·s^–1^)^46^ ,^58^ .

### Photosynthetic O_2_ Production and Respiration by Unicellular Algae

Photosynthetic organisms produce O_2_ in surplus when the intensity of photosynthetically active radiation (PAR) is above the light compensation point. A sufficient dose of light leads to an [O_2_] increase in the microalgal suspension, whereas darkness allows the monitoring of respiration. This offers a variety of experiments to answer diverse questions about the light compensation point, the interaction of photosynthesis and respiration, and the effect of specific inhibitors.

The O_2_-indicator to be used in combination with photosynthetic organisms must have an emission that is different from chlorophyll fluorescence emission. Therefore, we chose Pd-TPTBP, which emits around 800 nm where chlorophyll fluorescence emission is negligible.

The data displayed in Figure 12 show that the dose of light (i.e., amount of photons applied) determines whether a balance between photosynthesis and respiration is established or whether an O_2_ deficiency, approaching anoxia, occurs.

**Figure 12:**
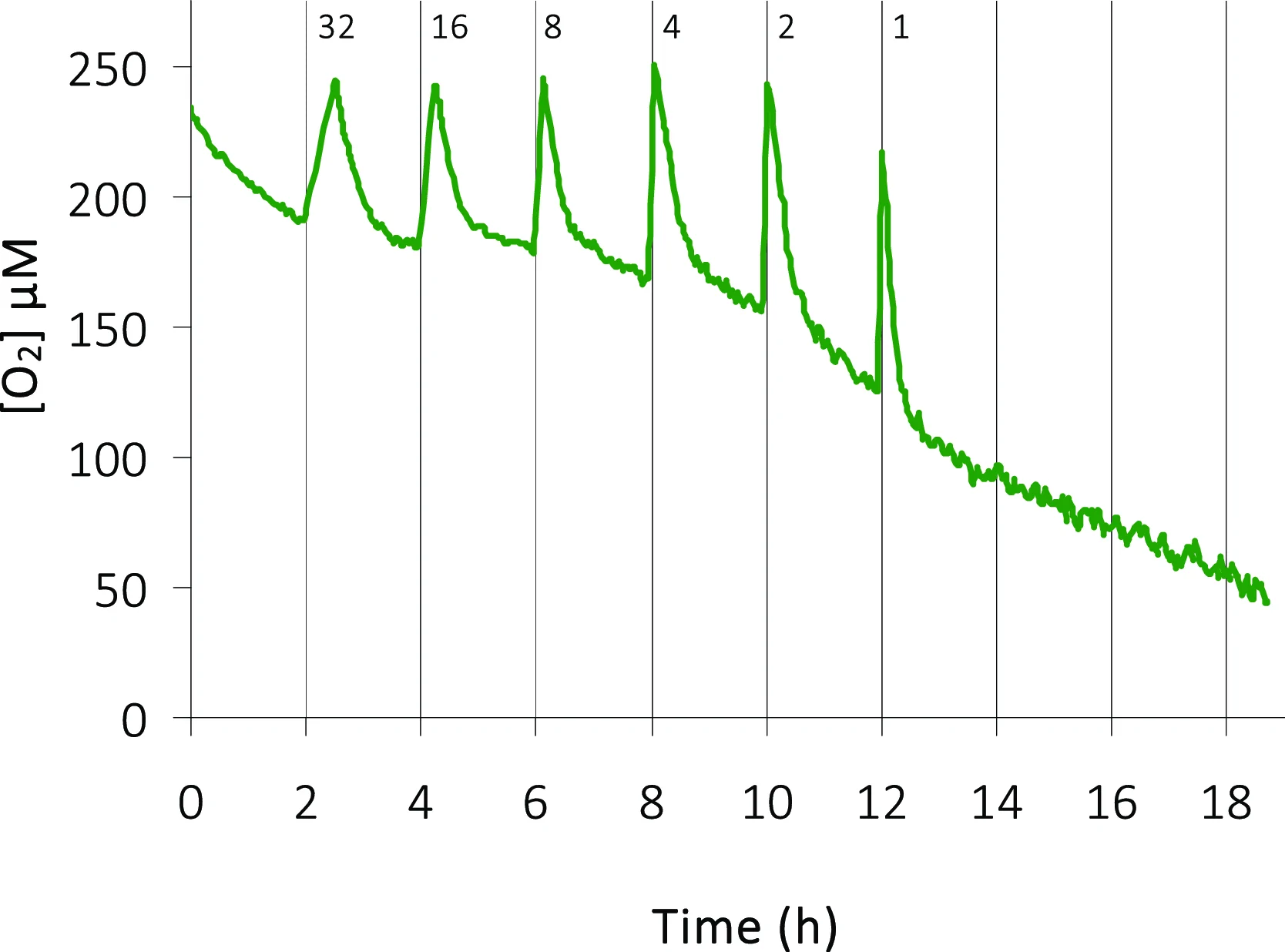
O_2_ consumption and production of O_2_ by microalgae (*Eremosphaera viridis*) monitored using phosphorescence from Pd-TPTBP (2) in foil-sealed MTPs. Respiration of algae was recorded for several hours. Every 2 h, the cells were irradiated with PAR (100 μmol·s^–1^·m^–2^) for several minutes. Numbers above each peak indicate the duration of irradiation in minutes. Variation of the light periods demonstrates that respiration cannot be compensated by less than 4 min of light. Experimental conditions: 200 μL algal suspension with ca. 14,000 cells per well; ^1^/_4_ M & S culture medium; 27 °C, 1.2 mS/cm; no salinity correction with calibration. Sample rate = 1/3·min^–1^.

Figure 13 demonstrates the inhibitory effect of 3-(3,4-dichlorophenyl)-1,1-dimethylurea (DCMU) on photosynthesis.^59–61^ The evolution of O_2_ during light periods is inhibited by DCMU (Figure 13; yellow trace). Since DCMU is soluble only in ethanol (EtOH), a control without DCMU was run in parallel, showing that EtOH has a pronounced stimulating effect on respiration (Figure 13; blue trace) when compared with the control (green trace). This latter effect is in line with earlier findings, showing that ethanol can serve as a carbon source for microalgae and stimulates metabolism and biomass production.^62,63^

**Figure 13:**
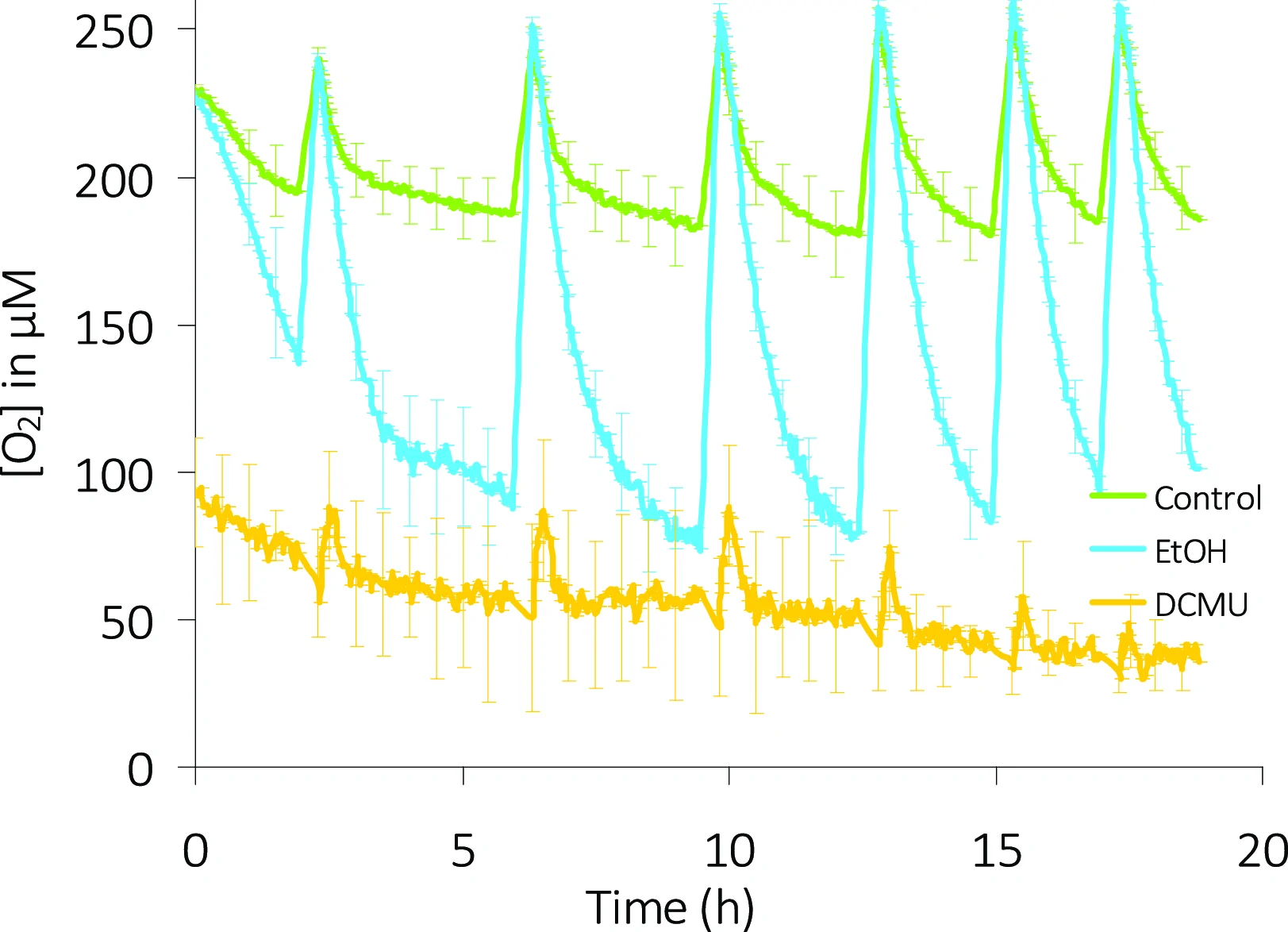
Effect of DCMU and ethanol on photosynthesis and respiration of microalgae (*Eremosphaera viridis*). Wells coated with Pd-TPTBP (2) were filled with 200 μL of algal suspension (∼7000 cell/ml) and sealed with adhesive, transparent polyester foil. [O_2_] was monitored in the absence of any inhibitor (green curve; “Control”), in the presence of ethanol (0.5%; blue curve), and in the presence of DCMU (100 μM), together with ethanol (yellow curve). Algae were irradiated at predetermined intervals with PAR of 25 μmol·s^–1^·m^–2^ for 20 min. AVR of *n* = 12; error bars represent SD. Experimental conditions: 1 M & S culture medium; 1.2 mS/cm; no salinity correction with calibration; 26 °C; sample rate = 1/3·min^–1^.

The experiment (Figure 13) has been repeated with diverse concentrations of DCMU and EtOH to roughly estimate the effective minimal concentrations needed for the effects seen (Supporting Information Figure SI 5 **:** Effect of DCMU and ethanol on photosynthesis and respiration of algal cells). A pronounced inhibition of photosynthetic O_2_ evolution can be seen with [DCMU] > 10 μM (Figure SI 5 B; yellow curve). Enhanced respiration is seen already with 0.03% EtOH (=6.8 mM) and increases further with increasing [EtOH] (Figure SI 5 A–F; blue curves).

### Oxygen Ingress Needs to be Kept in Check

O_2_ ingress and egress are undesirable effects when monitoring [O_2_] in MTPs, because the recorded phosphorescence should reflect only those [O_2_] changes that are attributable to the biochemical reactions in the MTP wells. Hence, these effects have to be kept in check, as demonstrated in the Supporting Information (Figures SI 7–SI 10). O_2_ ingress could be minimized to a negligible level by sealing the reaction volume from the atmosphere. Another strategy is to monitor O_2_ ingress separately, in order to correct for this effect when processing the experimental data. A procedure for quantifying oxygen ingress has been demonstrated above (Figure 7), and various measures have been proposed to reduce this effect.^34^ Among these was overlaying the reaction mixture with mineral oil. However, oil is permeable to O_2,_ and ingress is only slowed down, as demonstrated in the Supporting Information Figures SI 6 and SI 7. Wells containing solutions with different amounts of Na_2_SO_3_ were covered with different volumes of oil and O_2_ ingress was monitored. The data show that O_2_ ingress can be reduced by a reasonably thick layer of oil, but cannot be entirely prevented. This is in line with results presented by Arain et al. (2005).^34^

When sealing the MTP with an adhesive polyester film, a residual air volume with 21% O_2_ remains above the aqueous mixture to be probed in the well. This still causes unwanted O_2_ ingress, as demonstrated in the Supporting Information Figure SI 8. However, the data show that reducing the residual air volume in the wells reduces the O_2_ ingress considerably. There are diverse adhesive films and foils on the market that have been developed specifically to cover MTPs. We tested a few for their suitability to reduce or stop unwanted O_2_ ingress and found that polyester film, initially developed for sealing PCR reactions, is optimal. The film is transparent, allowing simultaneous OD_600_ and [O_2_] measurements (Figure 10), is largely impermeable to O_2_, and thus reduces O_2_ ingress to a negligible degree when the residual entrapped air volume is small.

### O_2_ Binding Capacity of the MTP Plastic Material

Polystyrene (PS) is known for its O_2_ permeability.^34^ O_2_ quickly diffuses into PS and quenches the phosphorescence of embedded porphyrins (Figure 11). Despite the low diffusion coefficient of O_2_ in PS, the effect is rapid since the indicator layer is thin (i.e., ≈1.5 μm). However, the whole MTP is made of PS and thus soaks up O_2_ like a sponge. Due to the absorption capacity of PS for molecular oxygen,^44^ bound O_2_ can diffuse out of the plastic material into the reaction, where [O_2_] is to be monitored.^64^ This effect is seen in the data demonstrated by Figure SI 10 in the Supporting Information

Wells of MTPs incubated overnight in an O_2_-free atmosphere in the presence of O_2_-absorbers were filled with Na_2_SO_3_ solution, and phosphorescence ratios were recorded for several hours. Two different effects can be seen (Figure SI 10, red dots). First, O_2_ is consumed by the sulfite oxidation reaction (Reaction 1) and the phosphorescence ratio increases within the first 40 min due to the [O_2_] decrease. Second, this process of O_2_ consumption is superseded by the diffusion of O_2_ from the ambient atmosphere into and through the O_2_-free MTP plastic material, leading to a ratio decrease and finally to an equilibrium at around *R* = 5. Control data (Figure SI 10, blue dots) show that O_2_ diffusing through and released from the O_2_-saturated plastic is largely consumed by the sulfite (Reaction 1), and the same equilibrium value is finally reached.

In summary, the O_2_ ingress cannot be completely avoided. However, there are means to minimize this interfering effect. An important factor is the time of the experiment. It is possible to estimate whether diffusion and ingress are negligible in comparison to the effect to be quantified (e.g., respiration or photosynthesis—Figures 12 and 13). Finally, appropriate controls run in parallel will allow for correction of the experimental data.

### pH Dependence of NMPs Entrapped in PS

The NMP phosphorescence is independent of the sample pH, as the indicator is trapped within the plastic material (PS) and thus changes of [H^+^] should not have an impact. This statement does not hold for any liquids in the wells because PS, when sulfonated, becomes proton-conductive.^65–67^

To demonstrate this effect, MTP wells doped with NMPs (1 or 2) were filled with buffers of different pH values, and phosphorescence was measured. Sulfonic acid buffers cause a significant pH dependence, revealing an H^+^-permeability of PS (Supporting Information Figures SI 11A, B and SI 12A,B; pH dependence of NMPs embedded in PS). Mineral buffers, in contrast, do not show this effect (Figures SI 11C,D and SI 12C,D). Hence, sulfonic acid buffer components in the reaction volume, such as MES, HEPES, and TAPS, make PS proton-permeable and will affect indicator phosphorescence.

## Advantages

Well-to-well variations of phosphorescence emission resulting from inhomogeneities in the indicator coating are eliminated by ratioing the phosphorescence intensities taken at two different wavelengths. The Pd-porphyrins 1 and 2 introduced here (Figure 1) are inherently ratioable indicators (Figure 5). In contrast to previous studies, no additional O_2_-insensitive reference dye is needed to obtain ratiometric phosphorescence emissions. With Pt-porphyrins and a coimmobilized O_2_-insensitive reference dye (e.g., rhodamine), a dynamic signal response factor of *R*_max_/*R*_min_ < 3 can be achieved.^21,38,39^ However, with Pd-porphyrins, which require no reference dyes, larger signal response factors are possible.

The indicator layers obtained with the coating protocol proposed here are clear and not turbid (Figure SI 1). This has the advantage that absorbance or optical density (OD) can be quantified simultaneously with [O_2_] in each well (Figure 10). The coating procedure is simple (see experimental section) and quick. Coating of 10 MTPs can be completed within 45 min, and the costs are below 5 € per plate (material only).

## Conclusions

Pd-porphyrins are superior O_2_-indicators for use in MTPs to monitor the concentration of oxygen dissolved in aqueous solutions. The novel coating protocol provided here allows inexpensive, fast, and easy production of MTPs for ratiometric optical readout. The conversion of the phosphorescence intensity ratios in terms of oxygen concentrations is straightforward. Thereby, the temperature and salinity effects can be taken into account. In summary, the MTPs prepared with immobilized NMP, as proposed in this study, meet all quality standards for spectroscopic sensors.^68^ The methods reported here open up a wide field of diverse research approaches, provided that critical interfering parameters are known and suitable control experiments are carried out.

## Experimental Section

### Specific Instrumentation and Materials

The plate reader Infinite M200 PRO with an injection unit (Tecan, Crailsheim, Germany) was used for optical readouts such as absorbance, optical density, and phosphorescence (bottom read). Suitable scripts were programmed via the menu interface of the manufacturer’s i-control software.

### Specific Chemicals

Chloroform, petroleum–benzine 40-60, palladium­(II) 5,10,15,20-(tetrapentafluorophenyl)-porphyrin (CAS 72076-09-6; *M*_W_ = 1079 g/mol), and palladium­(II) 5,10,15,20-(tetraphenyl)-tetrabenzoporphyrin (CAS 119654-64-7; *M*_W_ = 919 g/mol) were used. Sources and all other standard chemicals are listed in the Supporting Information (Table SI 1).

### Specific Consumables and Specific Disposable Material

Standard clear flat-bottom 96-well PS-MTP Sarstedt #82.1581. Aluminum paper sandwich film Carl Roth #X172.1. Transparent polyester sealing film Carl Roth #APT8.1. Standard consumables such as tubes, pipet tips, and dishes were from Sarstedt, Roth, or Greiner.

### Living Material

*Eremosphaera viridis* (de Bary) *V. natriegens* (*V*_max_)

### Buffers, Culture Media, Stock Solutions, and Coating Solutions

CPB (citric acid phosphate buffer) 50 mM citric acid adjusted with K_2_HPO_4_ to pH = 5.5. KPB (potassium phosphate buffer) 50 mM K_2_HPO_4_ adjusted with 50 mM KH_2_PO_4_ to pH = 7.4.

Algal culture medium: Murashige and Skoog medium (^1^/_4_ M & S): 1 g of MS salts plus 1 g of MES dissolved in 1 L of dH_2_O. The mixture was adjusted with KOH to pH = 6.2. Bacterial culture medium (LBv2): LB-medium supplemented with v2 salts (i.e., 204 mM NaCl, 4.2 mM KCl, and 23.14 mM MgCl_2_). PS stock solution 5% w/v in chloroform: 5 g of polystyrene (from Petri dishes) was dissolved in 100 mL of chloroform. Chloroform–petrol–benzine mix (CB-Mix 1:1): 100 mL of petroleum–benzine was mixed with 100 mL of chloroform.

TFPP stock solution: 1 mg of Pd-TFPP (1; M_W_ = 1079 g/mol) dissolved in 400 μL of PS-stock gives a deep purple-red color with about 2.3 mM of 1.

TPTBP stock solution: 1 mg of Pd-TPTBP (2; M_W_ = 919 g/mol) dissolved in 800 μL of PS-stock gives a rich green color with about 1.4 mM of 2.

TFPP coating solution: 400 μL of TFPP stock solution mixed with 11.6 mL of CB-Mix. The coating solution contains 51.7% chloroform (v/v), 48.3% petrol–benzine (v/v), 0.17% polystyrene (w/v), and 83.3 μg/mL TFPP (=77 μM)

TPTBP coating solution: 800 μL of TPTBP stock solution mixed with 23.2 mL of CB-Mix. The coating solution contains 51.7% chloroform (v/v), 48.3% petrol–benzine (v/v), 0.17% polystyrene (w/v), and 41.6 μg/mL TPTBP (=45 μM)

## Methods

### Coating Procedure

The coating is an improved and largely simplified procedure originally adapted from Borisov et al.^19^ and De Moraes Filho et al.^42^

From the coating solution, 30 μL is dispensed in each well of flat-bottom MTPs, and solvent vapors are evaporated overnight in a fume hood. This gives a homogeneous, clear, and transparent coating, as shown in the Supporting Information (Figure SI 1), with ca. 1 to 3 μg per well of NMP embedded in a PS layer of about 1.5 μm thickness.

Note: Coating solutions also depend on the type of MTP to be coated. Flat-bottom MTPs were preferably used here since they allow recording of OD_600_ and phosphorescence in parallel. For U-bottom MTPs, the coating solution should have a 3-fold concentration of porphyrin (i.e., 400 μL TFPP stock on 3.6 mL CB-Mix, or 800 μL TPTBP stock on 7.2 mL CB-Mix). Only 10 μL of the concentrated coating solution is needed to be dispensed per U-well to give a sufficiently homogeneous clear coating.

### Phosphorescence Recording

Wells of coated plates were loaded as described in the Results section. Phosphorescence and absorption readouts were recorded with the above-mentioned plate reader using specifically programmed i-control scripts.

### Calibration of NMP Phosphorescence Ratios in Terms of [O_2_] at Constant Temperature and Zero Salinity

A reasonable first approach for converting phosphorescence ratios R into oxygen concentrations [O_2_] is the assumption of a strict linear relationship (eq 5), provided the maximum O_2_ solubility [O_2_]_max_ is known together with the given salinity and temperature of the probed solution and the atmospheric pressure. *R*_max_ is the maximum phosphorescence ratio obtained under anoxic conditions ([O_2_] = 0 μM), whereas *R*_min_ is the ratio at maximum dissolved O_2_ in aqueous solution ([O_2_] = [O_2_]_max_).

5 $$\left[O_{2}\right]=\frac{R_{\max}-R}{R_{\max}-R_{\min}}\cdot {\left[O_{2}\right]}_{\max}$$

According to DOTABLES^3^ (Supporting Information Figure SI 2), [O_2_]_max_ is around 280 μM at RT = 20 °C, in fresh water (i.e., no salinity) and with normal atmospheric pressure of around 100 kPa.

### Accounting for Temperature Effects when Calibrating NMP Phosphorescence Ratios in Terms of [O_2_]

With increasing temperature θ (°C), the equilibrium O_2_ solubility (i.e., [O_2_]_max_) decreases in aqueous solutions.^2,69^ To account for this effect, the above calibration equation (eq 5) needs to be extended with a temperature-dependent term. Therefore, exponentials were fitted to data from dissolved oxygen tables (DOTABLES)^3^ to obtain continuous model functions describing the dependence of [O_2_]_max_ on temperature θ (eq 6; Supporting Information Figure SI 2).

6 $${\left[O_{2}\right]}_{\max}=A_{1}\cdot e^{-a\cdot \theta }+A_{0}$$

This term (eq 6) is used to correct for temperature effects when experiments were performed under varying temperatures. The resulting extended calibration equation is (eq 7)­

7 $$\left[O_{2}\right]=\frac{R_{\max}-R}{R_{\max}-R_{\min}}\cdot \left(A_{1}\cdot e^{-a\cdot \theta }+A_{0}\right)$$

For instance, the parameter triple of the model function (eq 6) fitted to the 100 kPa data set (Figure SI 2, red dots) is (A_1_; a; A_0_) = (323; 0.037; 126). The necessity of temperature correction is discussed and demonstrated in the Results section (Figure 8).

### Considering Salinity with Calibration

In addition to the temperature dependence, there is also a salinity dependence of oxygen solubility in aqueous media.^70^ [O_2_]_max_ decreases with increasing ion concentrations, which are typically expressed in units of specific conductivity (S/cm) of the solution. For more precise calibration, recorded [O_2_] needs to be corrected by a correction factor F_sal_ ^2^ to take account of the effect of salinity (eq 8).

8 $$\left[O_{2}\right]=\frac{R_{\max}-R}{R_{\max}-R_{\min}}\cdot \left(A_{1}\cdot e^{-a\cdot \theta }+A_{0}\right)\cdot F_{sal}$$

Values of *F*_sal_ for a series of salinities are shown in the Supporting Information (Figure SI 3) and can be obtained online from DOTABLES.^3^

A barometric correction was omitted, since all data presented in the context of this study were obtained at sea level, and weather-related changes in atmospheric pressure were neglected.

For experiments requiring calibration, two types of references were established.

(1) Wells were filled with 30 mg of O_2_-absorber powder (i.e., iron charcoal salt mix) each and sealed with adhesive polyester foil to obtain maximal phosphorescence and *R*_max_ at [O_2_] = 0.
(2) For saturation, i.e., to obtain minimal phosphorescence and *R*_min_ with [O_2_] = [O_2_]_max_, wells were filled with distilled water and left open for O_2_ exchange with the ambient air.

## Supplementary Material



## Notes

Appearance of transparent MTPs coated with Pd-Porphyrin in PS, flat-bottom MTPs for [O_2_] quantification, temperature dependence of oxygen solubility in aqueous solutions, equilibrium solubility of atmospheric molecular oxygen in water, salinity dependence of oxygen solubility in aqueous solutions, salinity correction factor F_Sal_, linear relationship between phosphorescence ratios and [O_2_], correlating NMP phosphorescence ratios with amperometric oxygen recording, effect of DCMU and ethanol on photosynthesis and respiration, effect of DCMU and ethanol on photosynthesis and respiration of algal cells, O_2_ ingress from ambient air, O_2_ ingress reduced with oil, effect of oil on the O_2_ ingress, O_2_ ingress from residual air volume entrapped under the sealing film, dependence of O_2_ consumption on residual entrapped air volumes, O_2_ ingress observed with different entrapped air volumes O_2_-diffusion into and through polystyrene, O_2_-absorption by and diffusion through the MTP plastic material, pH dependence of NMPs embedded in polystyrene, pH dependence of Pd-TFPP phosphorescence emission, and pH dependence of Pd-TPTBP phosphorescence emission (PDF)

## Abbreviations

1 = Pd-TFPP: Palladium­(II)-5,10,15,20-(tetrapentafluorophenyl)­porphyrin (CAS 72076-09-6)
2 = Pd-TPTBP: Palladium­(II)-5,10,15,20-(tetraphenyl)­tetrabenzoporphyrin (CAS 119654-64-7)
[M]: a molecular species M in brackets designates the concentration in the solution given
Avr: average
MW: molecular weight
a.U.: arbitrary units
OD: optical density
DCMU: 3-(3,4-dichlorophenyl)-1,1-dimethylurea
NMP: noble metal porphyrin
EtOH: ethanol
PAR: photosynthetic active radiation
fwhm: full width at half-maximum
PS: polystyrene
HTP: high throughput
SD: standard deviation
KPB: potassium phosphate buffer
SI: Supporting Information
MTP: microtiterplate

## Variable Declaration

τ (*Greek letter: tau*): time constant
R: Ratio of phosphorescence intensities
θ (*Greek letter*: *theta*): temperature
*T*: integration time
F: phosphorescence
*t*: time
